# Evolution of Metabolic Abnormalities in Alcoholic Patients during Withdrawal

**DOI:** 10.1155/2015/541536

**Published:** 2015-02-24

**Authors:** X. Vandemergel, F. Simon

**Affiliations:** Department of General Internal Medicine, Centres Hospitaliers Jolimont, 1400 Nivelles, Belgium

## Abstract

Chronic alcohol intoxication is accompanied by metabolic abnormalities. Evolution during the early withdrawal period has been poorly investigated. The aim of this study was to determine the evolution of metabolic parameters during alcohol withdrawal.* Patients and Methods*. Thirty-three patients admitted in our department for alcohol withdrawal were prospectively included.* Results*. Baseline hypophosphatemia was found in 24% of cases. FEPO4 was reduced from 14.2 ± 9% at baseline to 7.3 ± 4.2% at day 3 (*P* < 0.01). FEPO4 inversely correlated with albuminemia (rs = −0.41,* P* = 0.01). CPK level was 124 ± 104 IU/L in men and 145 ± 85 IU/L in women (nl < 308 and <192 IU/L, resp.), 7% and 28% of patients having a CPK level >nl, respectively. No correlation was found between the sodium and CPK levels (*P* = 0.75) nor between the CPK level and the amount of alcohol ingested (rs = 0.084, *P* = 0.097). Baseline urate level was elevated and returned to normal after three days. Baseline magnesium concentration was normal and stable over time.* Conclusion*. Chronic alcohol intoxication was accompanied by phosphaturia, rapidly reversible after alcohol withdrawal and inversely correlated with albuminemia, slight hyponatremia, low levels of 25 hydroxy vitamin D, elevated CPK level in about 30% of women, and hyperuricemia with rapid normalization.

## 1. Introduction

Alcohol consumption is associated with various metabolic abnormalities, including hypomagnesemia, hypophosphatemia, hypocalcemia, hypokalemia, metabolic acidosis, and respiratory alkalosis [1]. Regarding phosphate metabolism, chronic alcohol consumption induces hypophosphatemia by various ways: poor dietary intake of phosphate, gastrointestinal losses due to diarrhea, secondary hyperparathyroidism induced by vitamin D deficiency, or drugs interfering with phosphate absorption such as antiacids. Moreover, alcohol withdrawal is accompanied by acute respiratory alkalosis which can increase the hypophosphatemia as a result of an intracellular shift [2]. De Marchi et al. [3] have previously shown that excessive alcohol consumption is associated with a transient proximal tubular defect, another mechanism involved in hypophosphatemia found in these patients. Although cirrhosis seems to be associated with an increase in uric acid clearance [4], no other studies have reported a change in uric acid level in alcoholic patients without cirrhosis and during withdrawal. The aim of this study was to assess metabolic abnormalities and their short-term evolution and the change in fractional phosphate (FEPO4) and uric acid (FEUA) excretion in patients with excessive alcohol consumption without cirrhosis admitted to our department for alcohol withdrawal.

## 2. Patients and Methods

### 2.1. Patients

After having signed the informed consent, 33 patients admitted to the Department of General Internal Medicine (45 beds) for alcohol withdrawal were prospectively included and followed. Inclusion criteria were as follows: consuming large alcohol amounts for at least 6 months, drinking more than 3 glasses a day and having consumed alcohol in the last 24 hours, no histopathological evidence of cirrhosis or alcohol hepatitis, pancreatitis or malnutrition, no history of renal disease and diarrhea, and not having received medications known to influence renal function or mineral and electrolyte metabolism. The degree of alcohol consumption was assessed by self-report and family interview when possible. All patients were admitted voluntarily to alcohol withdrawal, thus minimizing the risk of underestimating the amount of alcohol ingested. The starting date of large alcohol consumptions was defined as the date of daily consumption of at least 3 glasses, every day. Alcohol consumption is expressed as grams of ethanol. Blood phosphate, sodium, chloride, creatine kinase, magnesium, uric acid, vitamin D, and creatinine levels were determined at baseline and 72 hours after withdrawal. Urinalysis of creatinine, phosphate, and uric acid levels was performed concomitantly. Biological markers of alcohol consumption such as the mean corpuscular volume and serum gamma-glutamyl transferase, aspartate aminotransferase, and alanine aminotransferase concentrations were measured in all patients. Routine biochemical determinations were performed in patient serum and urine using standard automated methods. When done, fibroblast growth factor 23 (FGF23) was measured using an ELISA kit (immunotopics). Forty subjects matched for age and sex were used as a control group and none of them was admitted for alcohol consumption.

### 2.2. Statistical Analyzes

Results are expressed as mean ± SD. A Student*t*-test was used. *P* values less than 0.05 were considered as statistically significant. Correlations were analyzed using a Spearman test.

## 3. Results

Thirty-three patients were included: 11 women and 22 men, with a mean age of 51 ± 14 years (range 29–80 years). In all patients, the mean alcohol consumption rate was of 194 ± 133 g per day. All patients had drunk in the last 24 hours before admission. Duration of large alcohol consumption was 108 ± 53 months and weekly alcohol consumption was 1358 ± 945 grams. Most patients had alcohol intoxication for many years.

**Figure 1:**
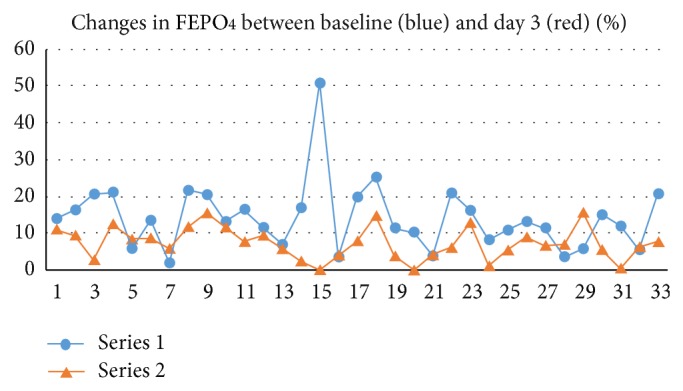
Changes in FEPO4 between baseline and day 3 after withdrawal.

Hypophosphatemia, defined as a plasma phosphate level less than 0.8 mmol/l, was found in 24% of patients at baseline and in 9% at day 3 after withdrawal. The baseline FEPO4 was 14.2 ± 9% and it dropped to 7.3 ± 4.2% at day 3 (*P* = 0.00038). Upon admission, 40% of patients had a FEPO4 >15% and one of them had a FEPO4 equal to 50%. At day 3, only 2 patients had a FEPO4 greater than 15% demonstrating that the phosphaturia was rapidly normalized. Changes in FEPO4 in the 33 patients are shown in Figure 1. The FEPO4 inversely correlated with the albuminemia (rs = −0.41, *P* = 0.01) but not with the mean corpuscular volume or with the vitamin D level.

Hyponatremia, defined as a sodium level ≤135 meql/l, was found in 18% of patients at baseline and it was normalized in all patients except one at day three after withdrawal. The change in sodium level was however not significant. No correlation was found between the natremia and the mean corpuscular volume (MCV). Creatine phosphokinase (CPK) level was 124 ± 104 IU/l in men and 145 ± 85 IU/l in women (nl < 308 and <192 IU/l, resp.), with, respectively, 7% and 28% of patients having a CPK level > nl. No correlation was found between the sodium and CPK levels (*P* = 0.75) or between the CPK level and the amount of alcohol ingested (rs = 0.084, *P* = 0.097). Comparing patients with and without CPK elevation, we did not find differences in terms of hypophosphatemia (0.93 ± 0.26 in the group with CPK elevation versus 1.04 ± 0.27 in the group without CPK elevation, *P* = 0.5) and fractional excretion of phosphate (resp., 17 ± 3.5% versus 14.8 ± 10%, *P* = 0.48). The mean albumin level was of 4.2 ± 0.4 mg/dL. An increased aminotransferase level >40 IU/l was found in 57% of cases. All patients except one had vitamin D deficiency upon admission and no change in vitamin D level was observed at day 3. No change in FEUA was observed during alcohol withdrawal but the urate level rapidly decreased to normal value. One of the patients had a very high phosphaturia (FEPO4 at 50%) and profound hypophosphatemia (0.29 mmol/l). In this case, the FGF23 level was measured and showed normal value (44.3 RU/mL; nl: 30–176).

Upon admission, all patients except one had a normal magnesium level which remained stable during alcohol withdrawal. The chloride plasma level was markedly increased while the bicarbonate level was unchanged. Biological parameters are reported in Table 1.

## 4. Discussion

Chronic alcohol consumption is accompanied by various metabolic abnormalities [5]. Elisaf et al. have previously shown that 22.8% of patients have hyponatremia, 31% have hypomagnesemia, and 29% have hypophosphatemia [5]. These results are in accordance with our own results except for the magnesium level.

We showed that hyponatremia resolved rapidly. Various mechanisms can lead to hyponatremia in alcoholism but they are related to hypovolemia in half of patients [6]. Animal studies have shown that acute alcohol injection is followed by an increase in creatine kinase level [7] and some studies have found that hyponatremia is sometimes accompanied by increased creatine kinase levels [8], two elements which can contribute to the increase in CPK level. In our study, however, only 7% of male patient had increased CPK levels at baseline. More women (28%) had an increased CPK level.

However, no correlation was found between the hyponatremia and the CPK level or between the CPK level and the amount of alcohol ingested. Previous studies have found an increased CPK level in 15% to 60% of alcoholic patients [9, 10]. Ethanol ingestion is toxic for muscle and leads to the rapid appearance of ultrastructural changes [11]. Furthermore, women seem to be at higher risk of alcoholic cardiomyopathy and myopathy than men [12]. In our study, the discrepancy in the percentage of patients with increased CPK level observed between men and women is a possible argument for this fact.

Hypophosphatemia, frequently observed in alcoholic patients, could be due to different causes [13] including malnutrition, vitamin D deficiency, diarrhea, or drugs interfering with phosphate absorption or an increase in phosphaturia. Regarding hypophosphatemia, phosphate diabetes is defined by a phosphate clearance >15 mL/min with a proximal tubular reabsorption rate <85% [14]. Upon admission, 24% of patients had hypophosphatemia, which is in accordance with the results found in the study by Elisaf et al. (29%) in 1994 [5]. Furthermore, upon admission, 40% of our patient had FEPO4 >15%. Phosphaturia did not correlate with the degree of vitamin D deficiency and it was not only related to vitamin D deficiency as the vitamin D level remained stable between baseline and day 3. Thus, the normalization of phosphaturia does not seem to be related to a change in vitamin D level. The mechanisms underlying the inadequate phosphaturia observed in alcoholic patients are not well understood but some authors have suggested that alcohol intoxication could induce proximal tubular abnormalities [3].

FGF23 is a bone-derived endocrine regulator of phosphate homeostasis which inhibits renal tubular phosphate reabsorption. No previous studies have focused on the relationship between the FGF23 level and the phosphaturia found in alcoholic patients. We only determined the FGF23 level in one case (with FEPO4 of 50%) which did not seem to be involved in alcoholic-induced phosphaturia. This hormone acts directly on renal proximal tubules to induce phosphaturia through activation of the ERK1/2-SGK1 signaling pathway [15]. No relationship between FGF23 and chronic alcohol intoxication is known and to our knowledge, there is no report on a role of the FGF23 level in case of alcohol-induced phosphaturia. An association between a moderate alcohol consumption and osteoporosis is plausible but conflicting results have been reported: some studies have shown an increased risk of fracture [16] while this risk is reduced in other studies [17]. However, in heavy drinkers, alcohol consumption is associated with a risk of osteoporosis [18]. It can be assumed that the degree of phosphaturia found in alcoholic patients could contribute to the development of osteoporosis but further studies are needed to confirm this assumption.

Moreover, increased serum uric acid levels are frequently found in alcoholic patients [19]. In our patients, the uric acid level rapidly decreased during alcohol withdrawal but no difference in FEUA was observed and no patient had a FEUA >15%. These results are not in accordance with those of De Marchi et al. who have shown that the serum uric acid level increased after withdrawal and that, upon admission, 11% of patients had increased FEUA >15%. No studies have focused on the change in FEUA during alcohol withdrawal. In 2002, Liberopoulos et al. [20] have reported the case of a patient admitted for severe hypouricemia (95.2 *μ*mol/l) with a FEUA of 26% and a moderate hypophosphatemia (0.45 mmol/l or 1.4 mg/dL) with a FEPO4 of 40%. Five days after alcohol withdrawal, the values returned to normal (phosphate level: 1.26 mmol/l or 3.9 mg/dL; FEPO4: 6%; uric acid level: 178.5 *μ*mol/l; FEUA: 12%). De Marchi et al. [3] have found no change in uric acid clearance during alcohol withdrawal except in 11% of patients in whom it was elevated. More studies are needed to understand the uric acid metabolism in alcoholic patients without cirrhosis. In conclusion, chronic alcohol intoxication is accompanied by an increased phosphaturia, ranging sometimes in the values of phosphate diabetes, which is rapidly reversible after alcohol withdrawal, even in the case of long-term consumption, and is inversely correlated with the albuminemia, a slight hyponatremia, an increased CPK level in about 30% of women, and the rapid normalization of the hyperuricemia.

## Figures and Tables

**Table 1:** Changes in biological parameters between baseline and day 3 after withdrawal.

	Baseline	Day 3	Control group
Plasma bicarbonate (mmol/L)	25.7 ± 3.8	26.03 ± 3.12 (NS)	26 ± 1.3 (NS)
Serum sodium (meq/L)	138 ± 4.1	140 ± 4.2 (NS)	137 ± 3.6
Serum chloride (meq/L)	96 ± 4.5	101 ± 4.5 (*P* = 0.0001)	98 ± 5.7
Serum phosphate (mmol/L)	1 ± 0.18	1.13 ± 0.23 (*P* = 0.09)	1 ± 0.23 (NS)
Serum magnesium (mmol/L)	0.78 ± 0.07	0.80 ± 0.1 (NS)	
Serum uric acid (mg/dL)	6.1 ± 1.7	4.9 ± 1.6 (*P* = 0.0007)	4.7 ± 1.6^*^
Vitamin D (ng/mL)	9 ± 6	10 ± 6 (NS)	
FEPO4 (%)	14.2 ± 9.06	7.3 ± 4.2 (*P* = 0.00038)	9.8 ± 4.4^**^
FEUA (%)	4.04 ± 3.3	4.36 ± 4.2 (*P* = 0.78)	7 ± 2.7^***^

^*^ *P* < 0.01 with serum acid uric at admission.

^**^ *P* < 0.005 with FEPO4 at admission.

^***^ *P* = 0.001 with FEUA at admission.
